# Monoclonal Gammopathy of Undetermined Significance Disguised as Chronic Neutrophilic Leukemia

**DOI:** 10.4084/MJHID.2010.002

**Published:** 2010-03-01

**Authors:** M.A. Hartley, L. Sokol, G. Caceres, M. A. Hussein, A. List, J. Pinilla-Ibarz

**Affiliations:** Hematology/Oncology Department, H. Lee Moffitt Cancer Center and Research Institute, Tampa, Florida 33612. USA

## Abstract

We encountered a 60-year-old woman with a medical history of diabetes mellitus, osteoporosis, peripheral vascular disease, and hypertension who had earlier presented at an outside facility with knee pain, which led to a finding of elevated neutrophil count of 35×10^9^/L. Because she was otherwise asymptomatic but continued showing elevated neutrophil levels, she sought a second opinion at our facility. Serum protein immunoelectrophoresis with immunofixation revealed an immunoglobulin A (IgA)-κ monoclonal gammopathy concentration of 1305 mg/dL (normal 80–350 mg/dL) but relatively normal concentrations of IgG of 840 mg/dL (620–1400 mg/dL) and IgM of 36 mg/dL (45–250 mg/dL). Using clonal analysis, we found a polyclonal expression pattern in all cell types analyzed. Comprehensive work-up for multiple myeloma and infectious etiology of neutrophilia was negative. We concluded that our patient’s neutrophilia may have been due to the underlying monoclonal gammopathy. This is the first case in the literature of a patient with monoclonal gammopathy of undetermined significance presenting with chronic neutrophilia, mimicking chronic neutrophilic leukemia (CNL). Patients with CNL have a poor prognosis; therefore, it is important to distinguish diagnostically between CNL and reactive neutrophilia.

## Introduction:

Monoclonal gammopathy of undetermined significance (MGUS), as defined by the International Myeloma Working Group, is a disorder characterized by a prominent monoclonal protein identified by serum electrophoresis. In MGUS, the serum monoclonal protein concentration is <30 g/L and the incidence of bone marrow clonal cells is <10% with no evidence of multiple myeloma. MGUS is thought to be a premalignant condition from which myeloma may arise; indeed, about 20% of patients with MGUS will develop myeloma. 1

Chronic neutrophilic leukemia (CNL) is an extremely rare entity, which is as yet not well defined. The main defining features are splenomegaly and a neutrophilic predominance in the peripheral blood, which cannot be explained by any infectious process or leukemoid reaction. In addition, the diagnosis of chronic myelogenous leukemia must be excluded. There should also be evidence of B-cell receptor (BCR) and ABL negativity.^2^ So far, no molecular markers specific to this disorder and no treatment that results in complete remission have been identified. The prognosis for patients is poor, given the very few therapies available for treatment, and the disease may become refractory to treatment. In addition, this disease has a strong potential for blastic transformation, which is often terminal for patients. Most treatment options for CNL, namely, hydroxyurea, busulfan, and 6-thioguanine, at best only stabilize the neutrophilia.

Researchers have observed an association between CNL and monoclonal gammopathies such as MGUS or multiple myeloma.^3^ Proposed theories for this association include the expression of high levels of cytokines, such as IL-6, produced by plasma cells, which may result in proliferation of other myeloid clones,^4^ and overproduction of colony-stimulating growth factor (G-CSF), which may induce myeloid growth.^5^ In this report, we present a unique case of MGUS with leukemoid reaction disguised as CNL.

## Case Report:

In December 2006, a 60-year-old female patient, non-smoker, with a medical history of diabetes mellitus, osteoporosis, peripheral vascular disease, and hypertension presented to an outside facility with complaints of right knee pain. An MRI of her knee showed abnormal bone marrow signaling. At presentation, complete blood cell counts were also made, showing white blood cell count of 44.11 × 10^9^/L, with a differential of 79% neutrophils, 4% lymphocytes, and no basophils. Her hemoglobin concentration was 14 g/dL and platelet count was 158 × 10^9^/L. At initial presentation, she had no other complaints; however, on review of systems, she did recall a 3- to 4-month history of subjective night sweats, along with constipation alternating with diarrhea. Leukocyte alkaline phosphatase score was elevated at 319 (normal 25–130). In addition, she had a mildly elevated γ-globulin level of 3.9 g/dL, as well as an elevated level of β-microglobulin at 3.1 mg/L. In January 2007, the patient’s peripheral blood underwent cytogenetic and FISH analyses for BCR and ABL; both tests were negative. PCR was also negative for BCR and ABL. Bone marrow biopsy for persistent neutrophilia showed overall hypercellularity, with plasma cell percentage 7% and normal cytogenetics. Computed tomography imaging of the chest, abdomen, and pelvis showed no significant abnormalities, including a normal spleen size at 11.3 cm. She remained asymptomatic with neutrophilia for several months. In July 2007, the patient was referred to our institution for a second opinion, given her persistently elevated neutrophil count (white blood cell count of 48.74 × 10^9^/L, with a differential of 92% neutrophils).

Repeat FISH and RT-PCR on the peripheral blood were both negative for BCR/ABL; JAK2-V617F mutation was also negative. Bone survey and spinal MRI were negative for findings consistent with multiple myeloma. However, results of a repeat β2-microglobulin analysis showed that levels were still elevated at 2.1 mg/L. Serum protein immunoelectrophoresis with immune-fixation revealed an IgA-κ monoclonal gammopathy concentration of 1305 mg/dL (normal 80–350 mg/dL) and relatively normal concentrations of IgG of 840 mg/dL (620–1400 mg/dL) and IgM of 36 mg/dL (45–250 mg/dL). Urine protein electrophoresis showed an M spike of IgA heavy chain with mild proteinuria. Extensive work-up for infectious process and a full screening evaluation for underlying occult neoplasm were both negative.

**Fig. 1.**
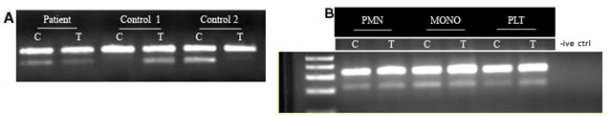
**(A)** Determination of iduronate-2-sulfatase (IDS) genotype. “C wells” contain polymerase chain reaction (PCR) products primed with C-specific oligonucleotide. “T wells” contain products primed with T-specific oligonucleotide. The patient is heterozygote for the polymorphism (both bands). Control 1 is homozygous for C allele, and Control 2 is homozygous for T allele (single band). **(B)** Clonality analysis. Total RNA, obtained from peripheral blood polymorphonuclear cells (PMN), mononuclear cells (MONO), and platelets (PLT), was transcribed into cDNA. Allele-specific PCR analysis of transcripts of IDS gene detected both alleles (C, T) in all analyzed cell types, suggesting polyclonal hematopoiesis. -ive ctrl = Negative control; simultaneously processed sample with substitution of H_2_O for DNA.

To determine the clonal origin of the hematopoietic cells in our patient, we employed a transcriptional clonality assay based on exonic polymorphism (C146T) of the X-chromosome-linked gene iduronate-2-sulfatase (IDS).^6^ Allele-specific PCR was used to detect the IDS gene polymorphism, by extracting and amplifying the DNA from the peripheral blood. Mononuclear and polymorphonuclear leukocytes were obtained by differential centrifugation with Lympholyte^®^–poly; platelets were obtained by high speed centrifugation of fresh plasma. Per methods developed by Gregg et al.,^6^ we amplified 3 ng of DNA using 250 pmol forward primer IDS1 and reverse primer IDS2R in 50 μL of PCR product with 1.25 Units iTaq polymerase (Bio-Rad, Hercules, CA) and reactant concentrations suggested for the enzyme. Five microliters of this first PCR product were used for the allele-specific reaction using the common reverse primer IDS2R and the specific forward primers, either IDS3C or IDS4T, in 50 μL of PCR product. The products were analyzed in 1.5% agarose gel with 0.4 μg/mL ethidium bromide to determine whether the sample was heterozygous for the polymorphism and informative for clonality. Because our patient was informative for this polymorphism (**Figure 1A**), we tested expression of the IDS allele in distinct hematopoietic lineages in peripheral blood. Using this PCR analysis, a polyclonal expression pattern was found in all analyzed cell types (granulocytes, mononuclear cells, and platelets) (**Figure 1B**).

Based on the polyclonality, we concluded that our patient’s neutrophilia was most probably secondary to the underlying monoclonal gammopathy. The patient remained asymptomatic; therefore, we opted to monitor her closely rather than initiate treatment.

## Discussion:

To date, there have been only 6 cases described in the literature of CNL associated with MGUS.^7^ This association may arise from a defect in the early hematopoietic stem cells, suggesting that this coexistence may be one continuous process, rather than two separate hematologic malignancies. In this case, the association is caused by the transformation of a pluripotent stem cell malignancy, which results in the proliferation of the two cell lines.^3^ Although yet to be proven, this theory could be applied to our case. Another explanation for the association between CNL and MGUS may simply be the propensity for older patients to develop multiple malignancies.

Other researchers^8^ have suggested that this phenomenon may be due to neutrophilic expansion as a response to cytokines released by plasma cells in patients with a monoclonal gammopathy.^4^ To support this theory, there have been reports that, during treatment of myeloma in patients with concurrent CNL (as clonal disease), the neutrophilia also resolves. 8,9 However, this theory fails to fully explain this relationship as some patients diagnosed with CNL have no concurrent diagnosis of monoclonal gammopathy, even after years.^10^ It also raises the question of why other cell lines were not also affected if this is a cytokine response.

In our patient, the possibility exists that the neutrophilia may be a precursor to or indicator of the eventual development of her monoclonal gammopathy. She does not fulfill the criteria for CNL as described. She lacks splenomegaly, and her neutrophils were a polyclonal population, not monoclonal. This suggests that the cells are non-neoplastic in nature since they arise from different pregenitors. In our case, one could postulate that the neutrophilia is related to the monoclonal gammopathy via an indirect stimulatory effect resulting in increased release of endogenous G-CSF. Studies have indeed shown the existence of high serum levels of G-CSF in patients with plasma cell neoplasm.^5^ Unfortunately, due to loss of follow-up, we were unable to obtain further blood samples from the patient to evaluate whether she had elevated serum cytokine levels of G-CSF or IL-6.

The polyclonality shown in our patient goes against a diagnosis of CNL.^11^,^12^,^13^ The confirmation of polyclonality indicates the absence of mutation in a pluripotent stem cell and also negates the clonal origin of neutrophilia and thus the diagnosis of CNL. One case report, by Katsuki et al.^14^, identified a patient in whom CNL transformed into an acute myeloblastic leukemia after 3 years. This resulted in death of the patient within 1 month, hence the importance of truly determining the disease entity as CNL versus leukemoid reaction resulting in neutrophilia.

Our case exhibits some similarity to that described by Stevenson et al.,^15^ in which a patient with IgA multiple myeloma and neutrophilia 1 year later developed myelofibrosis; however, in our case the neutrophilia may be secondary to IgA MGUS, not myeloma; in addition, our patient has no history of developing myelofibrosis. Based on the evidence that the neutrophilia in our patient could be reactive secondary to evolving plasma cell disorder, it is possible that eventual treatment of the MGUS could result in resolution of the neutrophilia.
